# Emerging frontiers in androgen receptor research for prostate Cancer: insights from the 2nd international androgen receptor Symposium

**DOI:** 10.1186/s13046-024-03125-5

**Published:** 2024-07-17

**Authors:** Justus Simon Israel, Laura-Maria Marcelin, Christian Thomas, Eva Szczyrbová, Susanne Fuessel, Martin Puhr, Johannes Linxweiler, Shivani Yalala, Wilbert T. Zwart, Aria Baniahmad, Jasper van Goubergen, Harri M. Itkonen, Adam Sharp, Edward O’Neill, Marc Pretze, Matthias Miederer, Holger H.H. Erb

**Affiliations:** 1 Department of Urology, Faculty of Medicine, University Hospital Carl Gustav Carus, Technische Universität Dresden, Dresden, Germany; 2 Department of Clinical and Molecular Pathology, Institute of Molecular and Translational Medicine, Faculty of Medicine and Dentistry, Palacký University and University Hospital, Olomouc, 779 00 Czech Republic; 3 German Cancer Consortium (DKTK), Partner Site Dresden and German Cancer Research Center (DKFZ), Heidelberg, Germany; 4 Department of Urology, Medical University of Innsbruck, Innsbruck, Austria; 5 Department of Urology, Saarland University, Homburg/Saar, Germany; 6 Department of Biochemistry and Developmental Biology, Faculty of Medicine, University of Helsinki, Helsinki, Finland; 7 Division of Oncogenomics, Oncode Institute, The Netherlands Cancer Institute, Amsterdam, The Netherlands; 8 Institute of Human Genetics, Jena University Hospital, Friedrich Schiller University, Jena, Germany; 9 Institute of Cancer Research, Sutton, Surrey UK; 10 MRC Oxford Institute for Radiation Oncology, Department of Oncology, University of Oxford, Oxford, UK; 11 Institut für Radiopharmazie, Helmholtz-Zentrum Dresden-Rossendorf, Bautzner Landstraße 400, D-01328 Dresden, Germany; 12 Department of Translational Imaging in Oncology, National Center for Tumor Diseases (NCT/UCC) Dresden: Faculty of Medicine and University Hospital Carl Gustav Carus, University of Technology Dresden (TUD), German Cancer Research Center (DKFZ) Heidelberg, and Helmholtz-Zentrum Dresden-Rossendorf (HZDR), Dresden, Germany; 13 Universitätsklinikum Carl Gustav Carus an der Technischen Universität Dresden, Fetscherstraße 74, 01307 Dresden, Germany

**Keywords:** AR, PCa, NR3C4, Androgen deprivation therapy, PSMA

## Abstract

Continued exploration of the androgen receptor (AR) is crucial, as it plays pivotal roles in diverse diseases such as prostate cancer (PCa), serving as a significant therapeutic focus. Therefore, the Department of Urology Dresden hosted an international meeting for scientists and clinical oncologists to discuss the newest advances in AR research. The 2nd International Androgen Receptor Symposium was held in Dresden, Saxony, Germany, from 26–27.04.2024, organised by Dr. Holger H.H. Erb. Following the format of the first meeting, more than 35 scientists from 8 countries attended the event to discuss recent developments, research challenges, and identification of venues in AR research. An important new feature was the involvement of PhD students and young investigators, acknowledging the high scientific quality of their work. The symposium included three covers: new advances from clinical research, basic and translational research, and novel strategies to target AR. Moreover, based on its increasing clinical relevance, a PSMA theranostic mini-symposium was added at the end of the AR symposium to allow the audience to discuss the newest advances in PSMA theranostic. This report focuses on the highlights and discussions of the meeting.

## Introduction

After a successful first “International Androgen Receptor Symposium “, the Department of Urology Dresden organised a subsequent symposium to provide experts in the androgen receptor (AR) and prostate cancer (PCa) field the opportunity to discuss the latest scientific advances and develop new research ideas [1]. The symposium occurred on April 26–27,2024, at the University Hospital Carl Gustav Carus (Dresden, Germany). Due to its clinical relevance, the symposium once more focused on the involvement of AR in PCa, the second most common cause of cancer-related deaths in men and was supplemented by a session highlighting recent advances in imaging and therapy towards PSMA [2, 3].

**Fig. 1:**
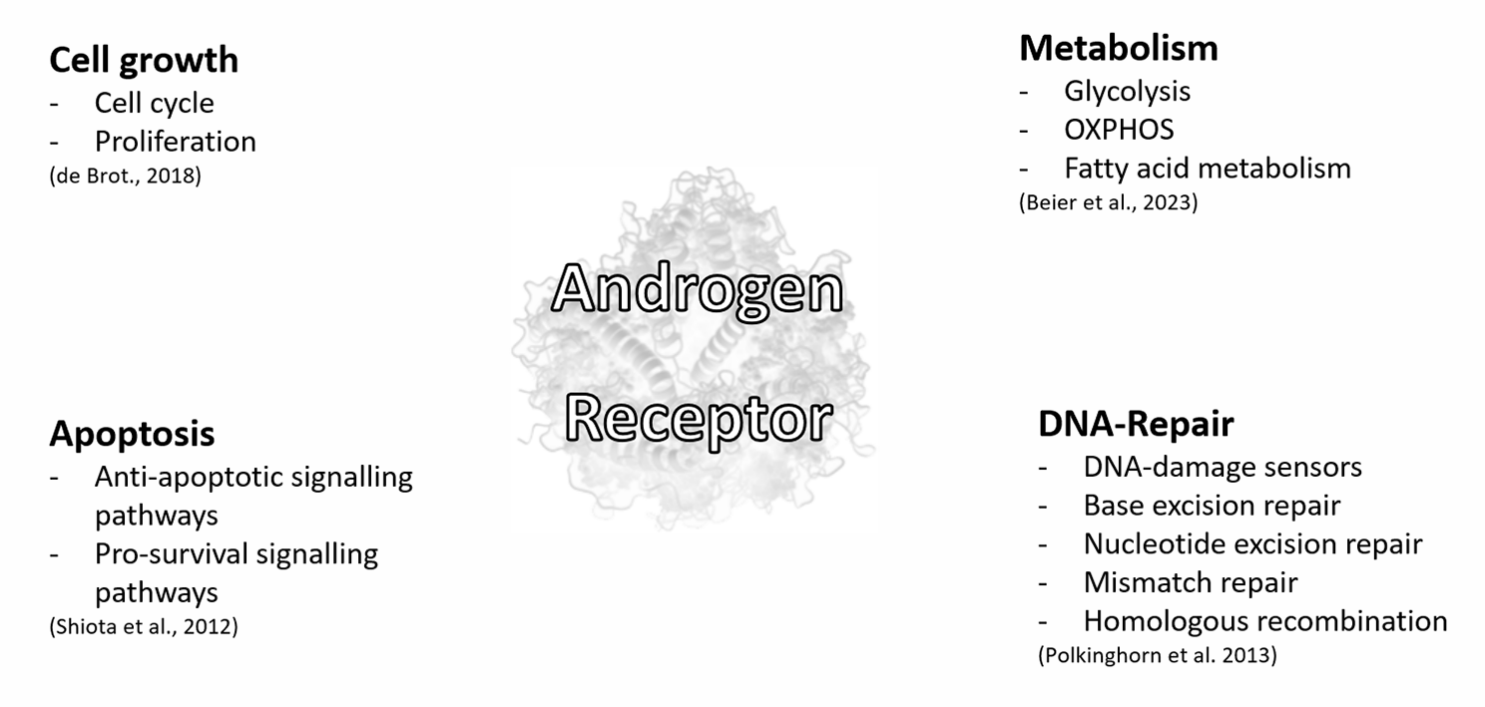
Overview of different roles of the androgen receptor in prostate cancer [5–10]

**Fig. 2:**
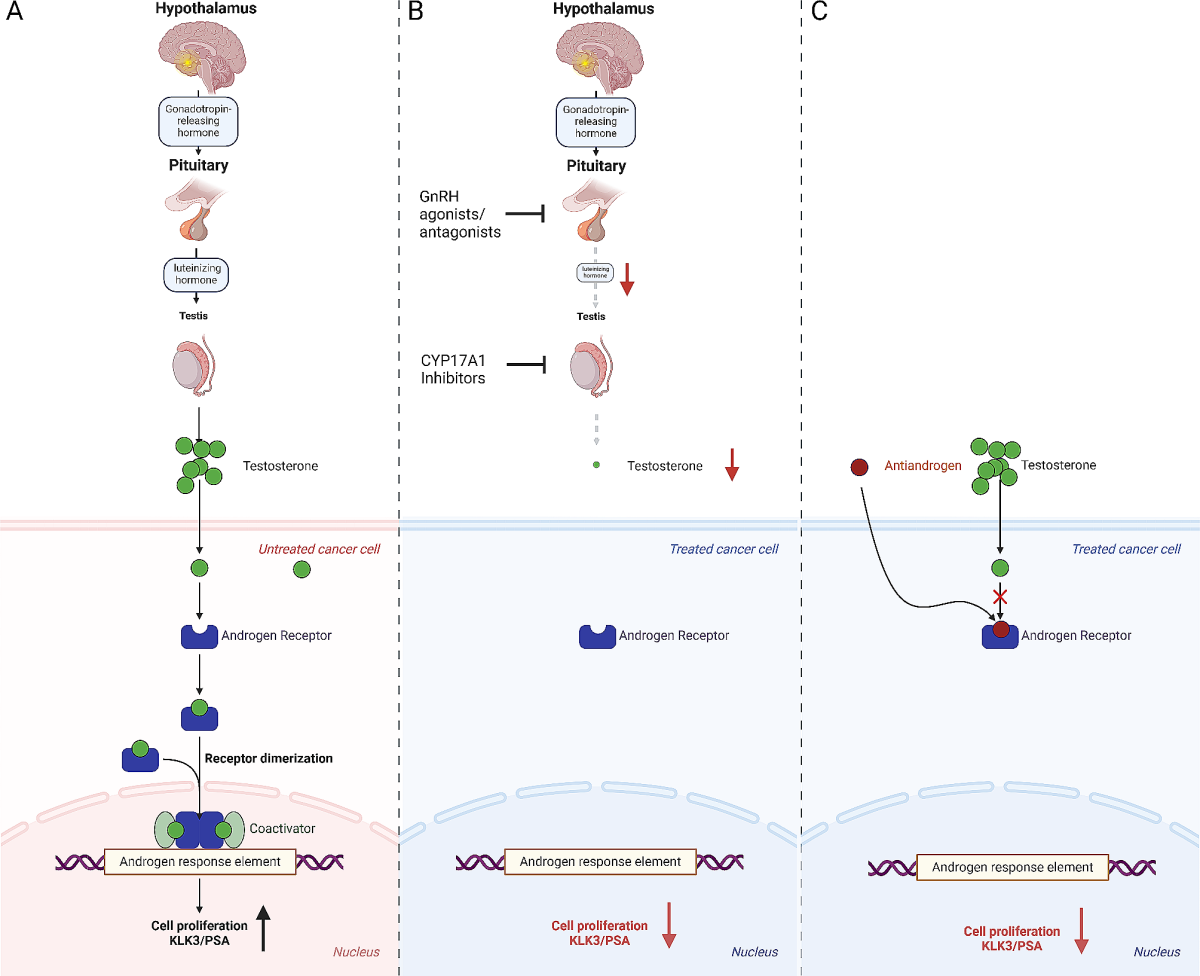
Simplified illustration of the various androgen receptor pathway inhibitors (ARPI) used. (**A**) The hypothalamic-pituitary axis controls the release and synthesis of androgens in testis and adrenal gland. These androgens are transported into the prostate tissue and converted intracellular to dihydrotestosterone (DHT). DHT subsequently activate the androgen receptor (AR), leading to the expression of AR-dependent genes, which play a significant role in the growth and survival of the prostate and prostate cancer (PCa). Key among these genes is the prostate-specific antigen (PSA), which serves as a biomarker for prostate health and disease progression. (**B**) During Androgen deprivation therapy (ADT), Gonadotropin-releasing hormone (GnRH) agonists, GnRH, agonists, and CYP17A1 inhibitors are used to reduce testosterone levels, as this hormone can stimulate PCa growth. GnRH agonists and agonists thereby aim to reduce the luteinising hormone, resulting in a reduction in testosterone production by the testes, mimicking the effects of surgical castration. CYP17A1 inhibitors directly inhibit androgen synthesis, leading to decreased circulating levels of androgens. Consequently, lower testosterone levels help slow down or shrink the growth of PCa cells. (**C**) Antiandrogens block the action of androgens at the androgen receptor (AR) level. These drugs block the AR’s ligand binding site, preventing androgens from attaching to and activating the receptor. Consequently, antiandrogens disrupt the androgen signaling pathway, which is crucial for the growth and survival of PCa cells. Illustration created by Biorender modifying the “Tamoxifen Mechanism of Action in Breast Cancer” template [97]

The development and function of the normal prostate and the progression of prostate cancer (PCa) hinge on androgens, requiring a continuous supply for cell growth and function [4–6]. The AR governs crucial processes such as differentiation, proliferation, DNA repair and metabolism (Fig. 1) in both [5, 7–10]. Localised tumours are curatively treated with radiotherapy or surgery, whereas metastatic cases rely on palliative pharmacological therapy [11, 12]. Given the pivotal role of the androgen signalling axis (Fig. 2A), therapies target AR activity through androgen deprivation therapy (ADT) (Fig. 2B) or antiandrogens (Fig. 2C), halt tumour progression and reduce tumour growth [10, 13]. However, despite the initial efficacy, treatment eventually faces resistance and disease progression, necessitating the development of new therapeutic approaches [14, 15].

### Session 1: New advances from clinical research

#### Treatment algorithms in mHSPC and mCRPC – from all in into precision medicine

Implemented by the astonishing results of the GETUG-AFU 15 study 10 years ago, the era of combination therapies has commenced clinical routine. It defines the new standard in treating metastatic hormone-sensitive PCa (mHSPC) [16]. Within the last decade, the therapeutic landscape for PCa has constantly evolved significantly, with new treatment options, including triple therapy, becoming available. Prof. C. Thomas reported in the opening presentation, “Treatment algorithms in mHSPC and mCRPC – from all into precision medicine”, on the current clinical challenges in PCa treatment and how new AR-focused combination treatments are implemented in the current treatment landscape. As ADT is the mainstay of mHSPC management, recent research has revealed its inadequacy as monotherapy in yielding optimal outcomes [17–19]. Studies have consistently revealed that the combination of ADT with AR pathway inhibitors (ARPI), such as enzalutamide, apalutamide, darolutamide, and abiraterone, is more effective than ADT monotherapy in avoiding disease progression and enhancing overall survival (OS) rates. No prospective randomised study is available for triple therapy (ADT + ARPI + Doc) vs. ADT + ARPI. Therefore, ADT + ARPI remains the standard treatment. Regarding meta-analyses, triple therapy might be an option for fit and chemotherapy-eligible patients with high-volume PCa may benefit from triple therapy with docetaxel [19]. Therefore, Prof. Thomas concludes that individualised decision-making between patients and their treating physicians remains essential due to the missing standard approach for all patients with mHSPC.

**Fig. 3:**
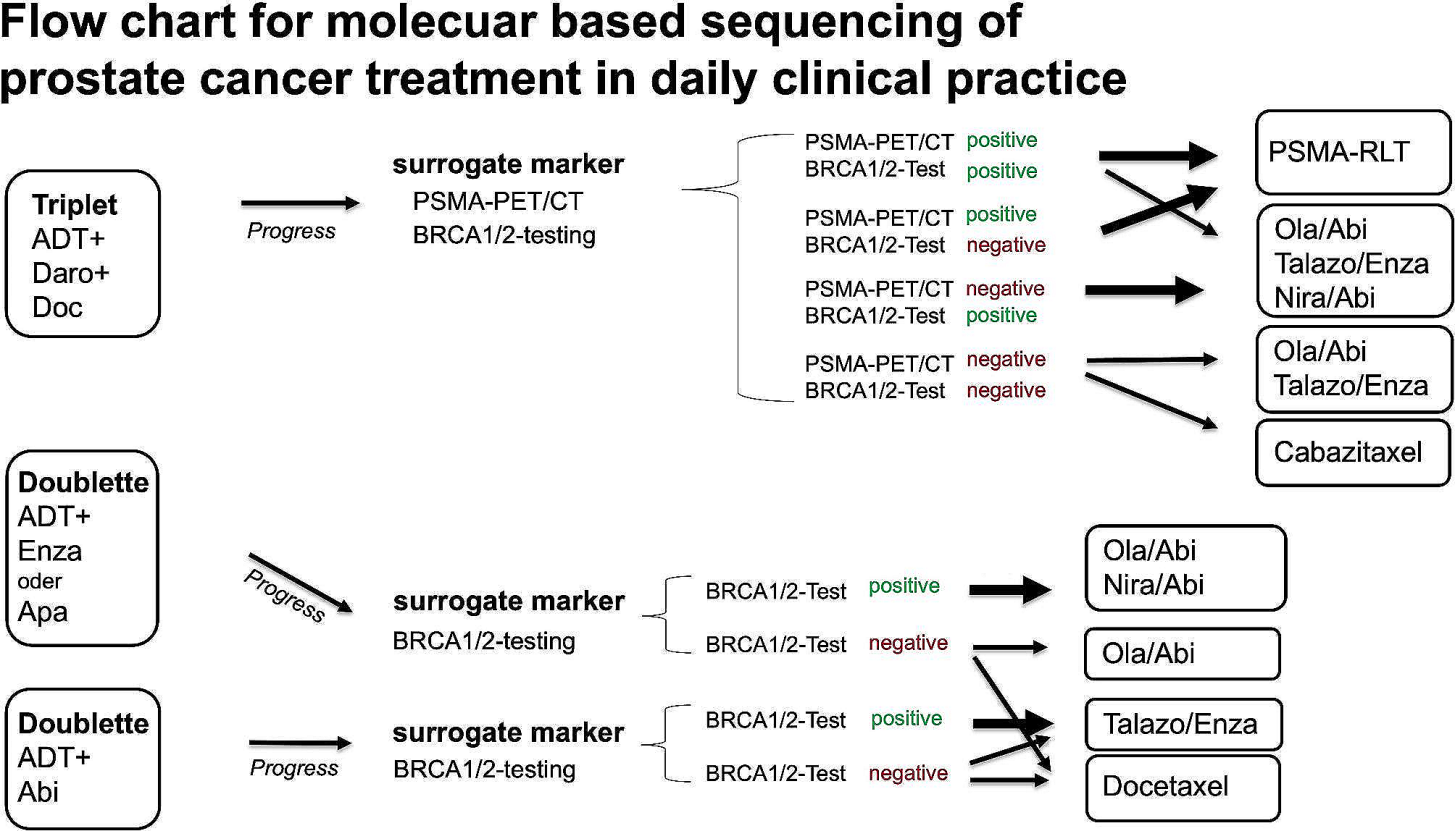
Flow chart for molecular-based sequencing of prostate cancer treatment in daily clinical practice as suggested by Prof. Thomas. Abberivations: ADT: androgen deprivation therapy, Abi: abiraterone, Apa: apalutamide, Daro: darolutamide, Enza: enzalutamide, Ola: Olaparib, PET: positron emission tomography, PSMA: prostate-specific membrane antigen, RLT: radio ligand therapy, Talazo: Talazoparib

Due to the change in the treatment landscape of mHSPC and the implementation of ARPI (Fig. 3) in a first-line setting, new strategies and treatment concepts are needed in castration-resistant prostate cancer (CRPC). In his talk, Prof. Thomas discussed the realisation of precision medicine in PCa. For example, he cited PARP inhibitor therapy, which has been approved in an all-comers concept combined with ARPI. Even though the results are promising, patients with pathological BRCA1/2 mutations respond better to the combination therapy [20]. Therefore, he highly encouraged molecular testing to improve therapy outcomes and new therapeutic strategies (Fig. 3). As a second example of precision medicine in PCa, he discussed the results of the TheraP and Vision trials, which justified PSMA-radio ligand therapy (RLT) as a therapy option in mCRPC [21–23]. Interestingly, the standard uptake value (SUV) seems to be a valid surrogate to predict treatment response [21, 24]. Therefore, he concluded that stratifying further third-line treatment using molecular imaging (PSMA-PET/CT) and molecular pathology (BRCA1/2) as surrogated would improve individualised precision medicine in PCa patients.

#### New advances of androgen receptor degraders

Targeted Protein Degradation (TPD) is an example of “from bench to bedside” science. The development of proteolysis targeting chimaera (PROTAC) has innovated the field significantly. These molecules hijack the ubiquitin-proteasome system and label target proteins for proteolysis [25]. In the second talk, Dr. Erb introduced the audience to specific AR-degraders ARV-110 and ARV-766, which are currently in phase I/II clinical trials (NCT03888612 and NCT05067140) [26–28]. The clinical trials for ARV-110 aimed for patients with heavily pre-treated mCRPC and recruited a total of 153 patients, of which ∼ 30% had AR ligand binding domain (LBD) mutations, including T878X/H875Y or L702H [26, 27]. The treatment was tolerated without difficulty, with no grade 4 treatment-related adverse events. However, the presence of the AR LBD mutation L702H diminishes ARV-110 efficacy according to the PSA_50_. As the AR L702H has a prevalence of 15% in untreated and up to 24% in treated mCRPC patients, this issue led to the development of ARV-766 (Luxdegalutamide), a second-generation PROTAC AR degrader, which overcame the L702H weakness and demonstrated a broader efficacy profile and better tolerability compared to ARV-110 in clinical settings [28–30]. Based on the current encouraging data, the clinical development of ARV-766 is likely to continue, and the PROTAC will ease into a phase III trial. However, Dr. Erb stated that as the second-generation ARV-766 targets the LBD, the constitutively active and clinically relevant AR splice variant (AR-V)3, AR-V7, and AR-V9 are not targeted and, therefore, may cause ARV-766 resistance and disease progress. Moreover, as LBD mutations seem to change the efficiency of LBD targeting PROTACs, new mutations may occur, diminishing the ARV-766 efficacy. Therefore, the PROTAC may be an option for a subgroup of patients with T878X/H875Y or L702H mutations. However, the current data indicates that only a subset of patients will likely benefit from the treatment. Therefore, the molecular profiling of the AR should be included before the treatment decision is made.

#### Analysis of the AR for clinical care of hormone-sensitive and castration-resistant PCa patients

Predicting therapy outcomes and monitoring therapeutic interventions using biomarkers is a powerful method for choosing the best therapeutic regimen and detecting emerging resistance at an early stage. Therefore, scientists and multicentre programs, such as the prostate biomarkers ProBio trial, are investigating new diagnostic and prognostic biomarkers [31]. In her MSc E. Szczyrbová’s talk, “Analysis of the AR for clinical care of hormone-sensitive and castration-resistant PCa patients”, she reported the importance and possibility of therapy monitoring by liquid biopsies. She reminded the audience that the right choice of biomarker is essential, and sample preparation time and type are also critical. Using her research as an example, she shows how possible markers for different stages of therapy should be tested. She presented possible validation cohorts with long-term follow-up data necessary to establish prognostic markers.

#### The long road of biomarker identification and validation: the RIBOLUTION project

In line with E. Szczyrbová, Prof. S. Füssel underlined the need for prognostic and diagnostic biomarkers. Her perspective emphasised the need to enhance PCa diagnostics, improve early prognosis systems for disease progression, and create tailored monitoring and precision therapies. Developing tools for prediction therapy response and identifying new therapeutic targets via molecular analyses is essential for personalised treatments and improved outcomes. Therefore, the interdisciplinary German research consortium RIBOLUTION, including Fraunhofer Institutes and universities, was established in 2011, primarily aiming to develop diagnostic and prognostic RNA tests for PCa. Using fresh frozen tissue specimens from radical prostatectomy explants and formalin-fixed paraffin-embedded biopsies, they created a prognostic transcript marker pattern comprising 1396 genes, the ProstaTrend Score [32]. The ProstaTrend Score represents, therefore, a potent prognostic RNA signature that has been discovered, with work ongoing to apply it to urine testing, which would be a much more readily accessible patient sample. These findings have undergone validation with different tissue types and endpoints and in various independent cohorts, even showing superiority to existing RNA signatures such as Prolaris, OncotypeDX, and Decipher. However, further validation in prospective studies and transferring these discoveries to the clinic remains a significant challenge.

### Session 2: New advances from basic and translational research

#### Glucocorticoid receptor (GR) signalling in the tumour-microenvironment - a clinically underestimated source for therapy resistance

**Fig. 4:**
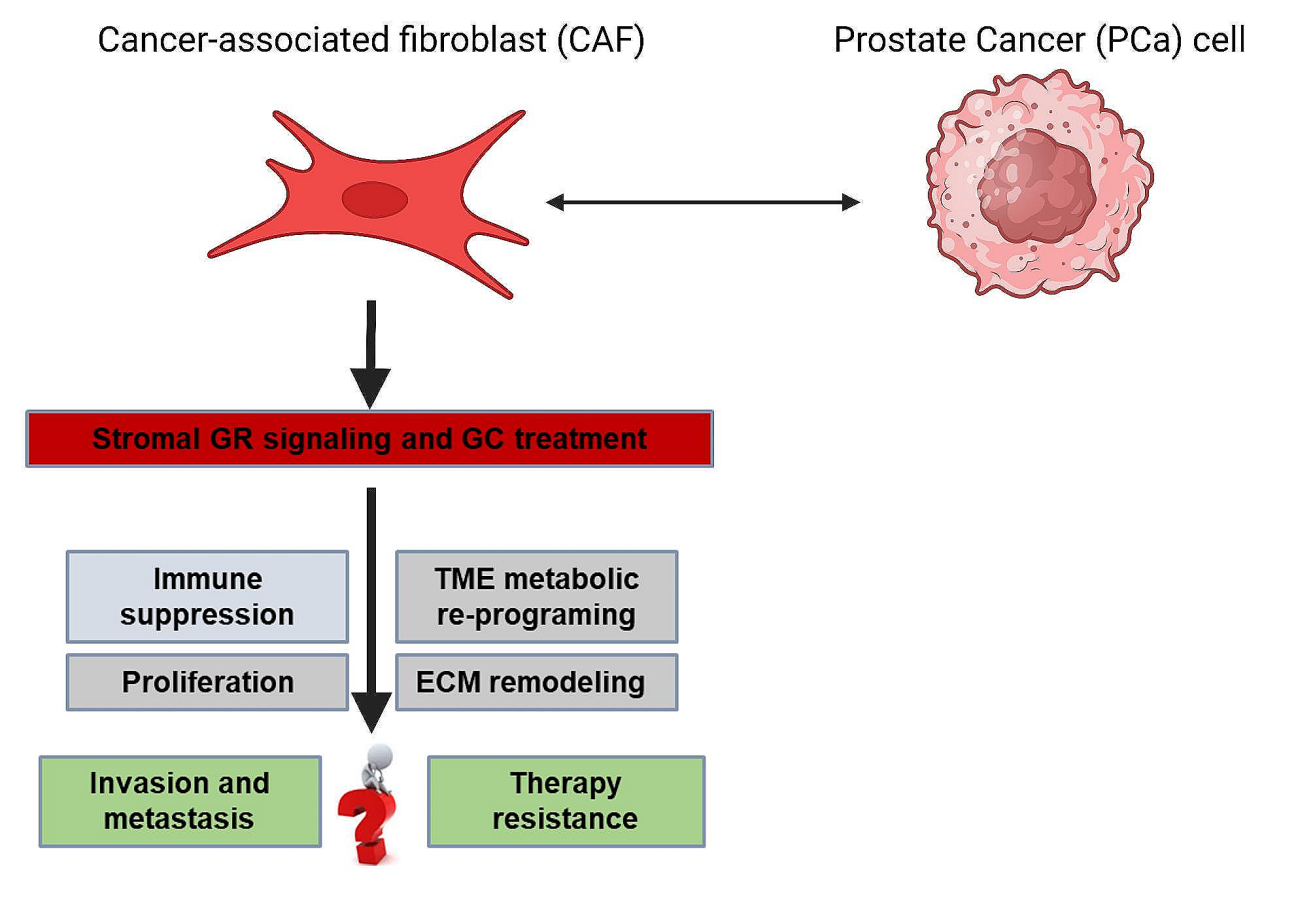
Graphic representation of Dr Puhr’s presentation of the role of the influence of glucocorticoids on the stroma in tumour progression [42, 98]. Illustration created by Biorender

Resistance to ADT and NHT is one of the most significant challenges in treating PCa [14, 15, 17]. Therefore, one of the main goals of basic and translational scientists in the AR field is to uncover resistance mechanisms and to identify potential therapeutic targets. Over the years, it has been demonstrated that AR over-expression, *AR* gene amplification, *AR* mutations/variants, and *AR* loss/neuroendocrine differentiation are possible resistance mechanisms against ARPI in PCa [14, 15, 17]. However, non-AR mechanisms have also been identified, such as the epithelial to mesenchymal transition or increased activity of transcription factors such as STAT5 and the GR [15, 33–36]. In CRPC, GR adopts the role of AR as a driver of cancer progression [37]. However, results from multiple clinical trials (NCT03674814, NCT03437941, NCT04033328, NCT03674814, NCT03437941) targeting both AR and GR failed to show benefit in patients with CRPC [38–41]. These trials indicate that although GR inhibition reduces cell growth in ARPI-resistant cells in vitro and in vivo, the situation seems more complex in CRPC patients [35, 37]. In Dr. M. Puhr’s talk, “GR signalling in the tumour-microenvironment - a clinically underestimated source for therapy resistance”, he presented his latest findings about the GR in PCa and his view on the role of the GR in ARPI resistance (Fig. 4). He revealed that GR activation increases stromal GR signalling, altering gene expression, protein levels, cancer-associated fibroblast (CAF) ‘s morphology, and increased protein secretion such as Interleukin (IL-)8 and IPTG [42]. Consequently, cell growth, colony formation, and 3D-spheroid processes of PCa epithelial cells are affected [42]. Furthermore, alterations in the adhesion-related proteins of CAFs following GR activation result in extracellular matrix remodelling [42]. Therefore, glucocorticoid-mediated GR signalling affects the CAF secretome and extracellular matrix architecture [42]. Consequently, these CAFs could counteract the treatment regimens and should be included as a therapeutic target structure in concomitant glucocorticoid therapy.

#### Modelling prostate cancer in mice with special emphasis on tumour-microenvironment

Due to the better understanding of PCa and its interaction with the tumour microenvironment, there is a need for more clinically relevant models of PCa. Therefore, Dr. Linxweiler provides an overview of the advantages and disadvantages of different PCa in vivo models. In his opinion, the essential requirements for PCa in vivo models are that they accurately simulate the natural disease progression (including local progression and metastatic spread), adequately represent tumour heterogeneity and the tumour microenvironment, demonstrate high reproducibility (with stable growth and high take rates), and are user-friendly. Therefore, he presented the advantages, disadvantages and pitfalls of xenograft models, patient-derived xenografts (PDX), and genetically engineered mouse models. In the first part of his talk, he compared sub-cutaneous with orthotropic PCa xenograft models. Even if the orthotropic xenografts model is more technically demanding and requires sophisticated equipment for tracking tumour growth, he believes that due to the higher engrafting rates, the natural routes of metastatic spread, and the well-vascularised microenvironment is the more relevant model more superior and clinically relevant PCa xenograft model [43]. He sees a better representation of tumour heterogeneity and the tumour microenvironment (TME) of the PCa in the derived PDX models. However, these models have only stable growth in a low percentage of cases (10–40%), and the risk of spontaneous development of Epstein-Barr Virus-associated lymphomas makes the PDX models highly challenging [44, 45]. However, these PCa xenograft models have significant drawbacks because they are implanted in highly immunocompromised mice. This problem can be avoided by using genetically engineered by using genetically engineered or syngeneic mouse models. These models allow researchers to observe the progression of PCa from precursor lesions to metastatic disease, studying specific molecular changes along the way [46]. However, while the murine immune system is functional in these models, they do not capture the full molecular complexity of PCa or adequately represent its heterogeneity and TME. Based on his experience, he concludes that there is a high demand for PCa mouse models for research purposes. Numerous models exist, each with weaknesses and strengths, and choosing the most appropriate one depends on the specific scientific question being addressed. In vivo mouse models remain crucial for this field, with stable growing PDX models and humanised models emerging as promising areas for future research.

#### What if…? – changes in the AR protein level are the main regulator of its activity

**Fig. 5:**
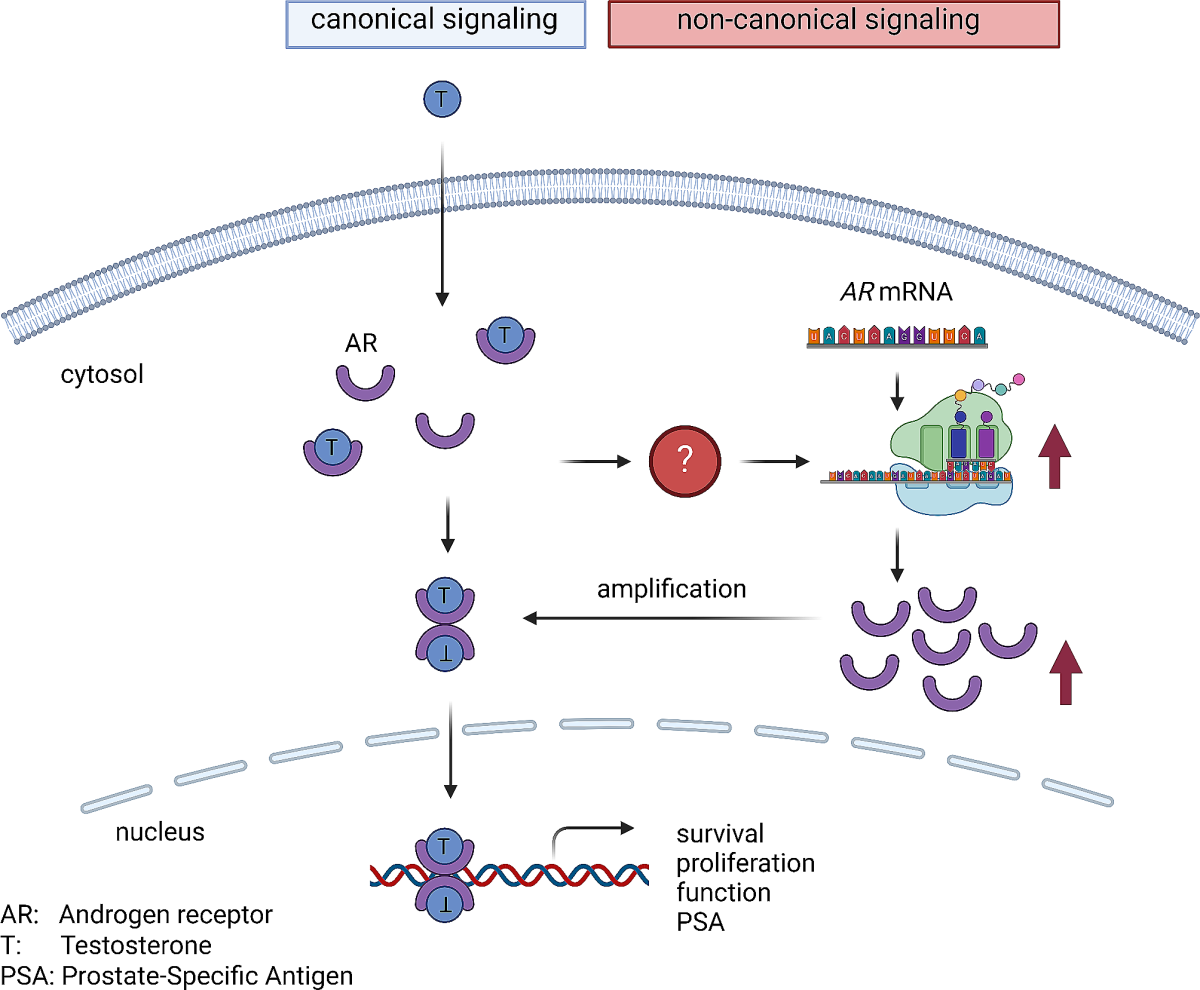
Graphical representation of the hypothesis by J. Israel and L. Marcelin on the role of translation in the androgen receptor signaling pathway. Abberivations: AR: androgen receptor, T: testosterone, PSA: prostate-specific antigen. Illustration created by Biorender

Dysregulated AR activity is involved in several pathological conditions, including PCa. In PCa, AR impacts tumour initiation and progression. Consequently, antagonising AR-activity via ARPI is an indispensable treatment strategy in metastasised PCa. In their talks, the medical doctor candidates, Ms. L. Marcelin and Mr. J. Israel, hypothesised the possibility that the regulation of the AR protein is an essential regulator of AR activity. Based on the observation that competitive binding of antiandrogens to the AR leads to a decrease in AR protein levels, they reported that the extent of AR reduction following antiandrogen treatment indicated the treatment response [47, 48]. As this change in AR protein was not linked to changes in the *AR* mRNA, they hypothesised that proteasomal degradation is responsible for AR reduction after antiandrogen treatment. As proteasomal inhibitors could not rescue this reduction in AR after antiandrogen treatment, they tested the involvement of the translational machinery, as previous studies suggested that androgen-induced AR protein increase depends on translation [47, 49]. Their preliminary data revealed that the inhibitor of translation, cycloheximide, reduces AR protein levels and AR activity comparable to enzalutamide, even in the enzalutamide-resistant PCa cells. They concluded that regulating AR protein is a vital regulator of AR activity (Fig. 5).

#### Androgen receptor action in time and space

In the final talk on the first day, Prof. W. Zwart explained his view on the formation of the AR transcription complex and when and where the different parts bind to the DNA in the cell nucleus. Moreover, he discussed whether epigenetic analyses in clinical trials can be used to predict response to enzalutamide treatment. Deviations in cancer cell’s epigenetic landscape are critical drivers of PCa tumourigenesis and progression [50]. These reversible epigenetic modifications represent an attractive and exciting novel target for treatment strategy against PCa. Curiously, one would indirectly affect AR signalling by affecting the epigenetic landscape. Using specimens from clinical trials to study epigenetic plasticity, they could show that enzalutamide induces epigenomic plasticity towards pro-survival signalling and uncovered the circadian regulator ARNTL as an acquired vulnerability after AR inhibition [51]. Therefore, he concluded that epigenetic profiling after treatment could reveal epigenetics-based biomarkers for response prediction and offer the opportunity for new synergistic drug combinations.

### Session 3: Novel strategies to target the androgen receptor

#### Ligand-specific protein composition of exosomes derived from treated prostate cancer cells

This session focused on novel strategies to target AR signalling to facilitate new possibilities for inhibiting AR-mediated PCa progression, even in ARPI resistance. It has been established that androgens can exhibit both tumour-promoting and tumour-suppressive effects [49, 52, 53]. A biphasic growth response showed that while normal levels promote growth, supraphysiological androgen levels (SAL) can reduce growth in AR-expressing PCa cells. However, the exact mechanism behind SAL’s repressing effects has not yet been described. In his talk, Prof. A. Baniahmad revealed that SAL can induce cellular senescence in CSPC and CRPC in preclinical PCa models to the same extent as antiandrogens [54]. Data from his research group showed that antiandrogens and SAL induce a tumour suppressive program by the p15/p16 – pRb – E2F1 – Cyclin D1 axis [54, 55]. However, he reported a SAL-activated increase in CD9 levels, indicating an enhanced exosome secretion. This result suggested that SAL treatment specifically alters exosome protein content, offering insights into the AR regulation of exosomal proteins. These exosomes subsequently promoted the growth of LNCaP cells, underscoring their potential tumour-promoting activity within the microenvironment. This study enhances our understanding of AR-regulated exosome secretion by AR-ligands and how their protein content can mediate tumour growth [54].

#### Targeting the CLK2/SRSF9 splicing axis leads to decreased AR-V7 expression in prostate cancer

Alternative splicing of *AR* mRNA produces the AR-V7 splice variant, a currently undruggable resistance mechanism to ARPI [14, 15]. AR-V7 lacks the ligand-binding domain targeted by hormones and antiandrogen antagonists, yet it continues to activate AR signalling. MSc. J. van Goubergen’s presentation, “Targeting the CLK2/SRSF9 splicing axis in PCa leads to decreased AR-V7 expression in an rs5918762 allele-dependent manner”, specifically addressed and circumvented this issue. In a previous study, Protein kinase C-β (PKCβ) was revealed as a druggable regulator of transcription and splicing at the AR genomic locus. Melnyk and colleagues revealed that targeting PKCβ was identified as an approach to repress *AR* genomic locus expression, including AR-V7 [56]. Through PKCβ inhibition, total *AR* gene expression was reduced, parallel lower AR-V7 protein levels and increased sensitivity of PCa cells to ARPI. Following a similar idea, MSc. J. van Goubergen discussed that the serine and arginine-rich splicing factor 9 (SRSF9)-CDC-like kinase (CLK)2 axis is identified as a clinically relevant target for therapeutic intervention. Moreover, inhibition of CLK leads to modifications within the tightly regulated SRSF9-AR-V7-CLK2 axis, suggesting the potential for combination therapies to achieve synergistic effects [57, 58]. However, ARPI-resistant PCa cells with a more mesenchymal phenotype demonstrated reduced sensitivity to CLK [59]. These findings highlight new avenues for targeted treatment strategies in PCa.

#### Targeting the transcription machinery to control PCa

A significant fraction of PCa cells can circumvent the AR-targeted therapies and go on to activate a pro-survival transcriptional program despite the presence of the therapy. One way to effectively target this program is to look into the key players maintaining high levels of transcription in the PCa cells, cyclin-dependent transcriptional kinases (CDK). CDKs 7, 9, and 12 phosphorylate RNA polymerase II during transcription initiation, release from promoter-proximal pausing, and sustain phosphorylation on the long genes, respectively [60]. Curiously, *CDK12* inactivation characterises an aggressive sub-type of PCa [61], and potential acquired sensitivities of these mutant cells are currently under intense investigation.

In his talk, Dr Itkonen discussed the importance of measuring the nascent transcriptome using tools such as SLAM-seq rather than the overall transcriptional program when establishing direct causal effects [62]. Using SLAM-seq, he demonstrated how even the short-term inhibition of CDK12 activity increases transcription of the short genes at the expense of the long genes. The transcriptional defects resulting from decreasing CDK12 activity lead to a generation of the ligand-independent forms of AR, as previously reported by Sun & al. [63]. Furthermore, Dr Itkonen showed that inactivation of *CDK12* results in acquired sensitivity to otherwise non-essential regulators of the spliceosome, including Serine/arginine-Rich Splicing Factor protein kinase-1 (SRPK1) [64, 65]. In his talk, he showed that SRPK1 can be targeted using Endovion, a compound currently in clinical trials against other cancers.

MSc Yalala presented data to show that CDK9 inhibition results in the downregulation of most of the genes but activates a selective set of genes related to an inflammatory response. She showed that AT7519, a CDK9 inhibitor tested in multiple clinical trials against other tumour types [66, 67], activates an innate immune response in PCa cells. These experiments were motivated by an earlier notion that CDK9 inhibition causes excessive splicing defects and results in transcriptional signatures of antigen presentation [68–70]. MSc Yalala explained that CDK9 inhibition changes gene transcription and triggers an inflammatory response by causing splicing defects that activate the double-stranded RNA (dsRNA)-activated protein kinase R (PKR), leading to NFκΒ signalling and selective transcription of the genes related to the innate immune response [71]. These effects were observed in the androgen-deprived conditions, which are known to increase the activity of the major oncogene, MYC, in PCa cells [72]. MSc Yalala then demonstrated that hyper-activation of MYC augments the immunogenic signalling induced by CDK9 inhibition. In aggregate, she concluded that similar to viral infection, CDK9 inhibition downregulates overall transcription but selectively activates part of the genome, particularly the genes of the innate immune response. These results propose that CDK9 inhibitors enhance the efficacy of immunotherapy.

#### Targeting persistent androgen receptor signalling in lethal prostate cancer

In the final talk of this session, Dr. Sharp explained his personal bias and daily thoughts driving his research interest. He emphasised the importance of understanding disease biology to identify relevant patients and develop targeted therapies based on biological mechanisms. In particular, he highlighted the importance of converting biological insights into positive predictive biomarkers to enhance diagnostic and treatment precision. He conducts clinical studies to test the proof of mechanism and concept, ensuring scientifically sound therapies tailored to individual patient needs, ultimately improving clinical outcomes. Based on his discoveries and literature, Dr Sharp is convinced that AR remains a critical target for advanced PCa. However, nearly all patients eventually develop treatment resistance due to persistent AR signalling through various mechanisms, highlighting an urgent need for therapies that can halt this response. He introduced the audience to mechanisms such as AR aberrations, deleterious myeloid cells, and bacterial androgen synthesis, causing persistent AR signalling in late-stage PCa [73–76]. However, finding the right therapy that halts the persistent AR signalling is a current remarkable challenge in offering customised therapy. He discussed the potential of targeting AR co-regulators such as heat-shock proteins (HSP) in lethal PCa to overcome persistent AR signalling. HSPs are important in AR stability, activity, and splicing, therefore representing an exciting target to limit AR signalling [77–79]. However, none of the developed drugs so far have made it from the bench to the bedside yet [80]. He hopes that new drugs will be developed soon and encouraged the audience to ask whether the drugs work and to include studies that provide ‘proof of mechanism’ within clinical studies.

### Session 4: PSMA theranostics mini-symposium

**Fig. 6:**
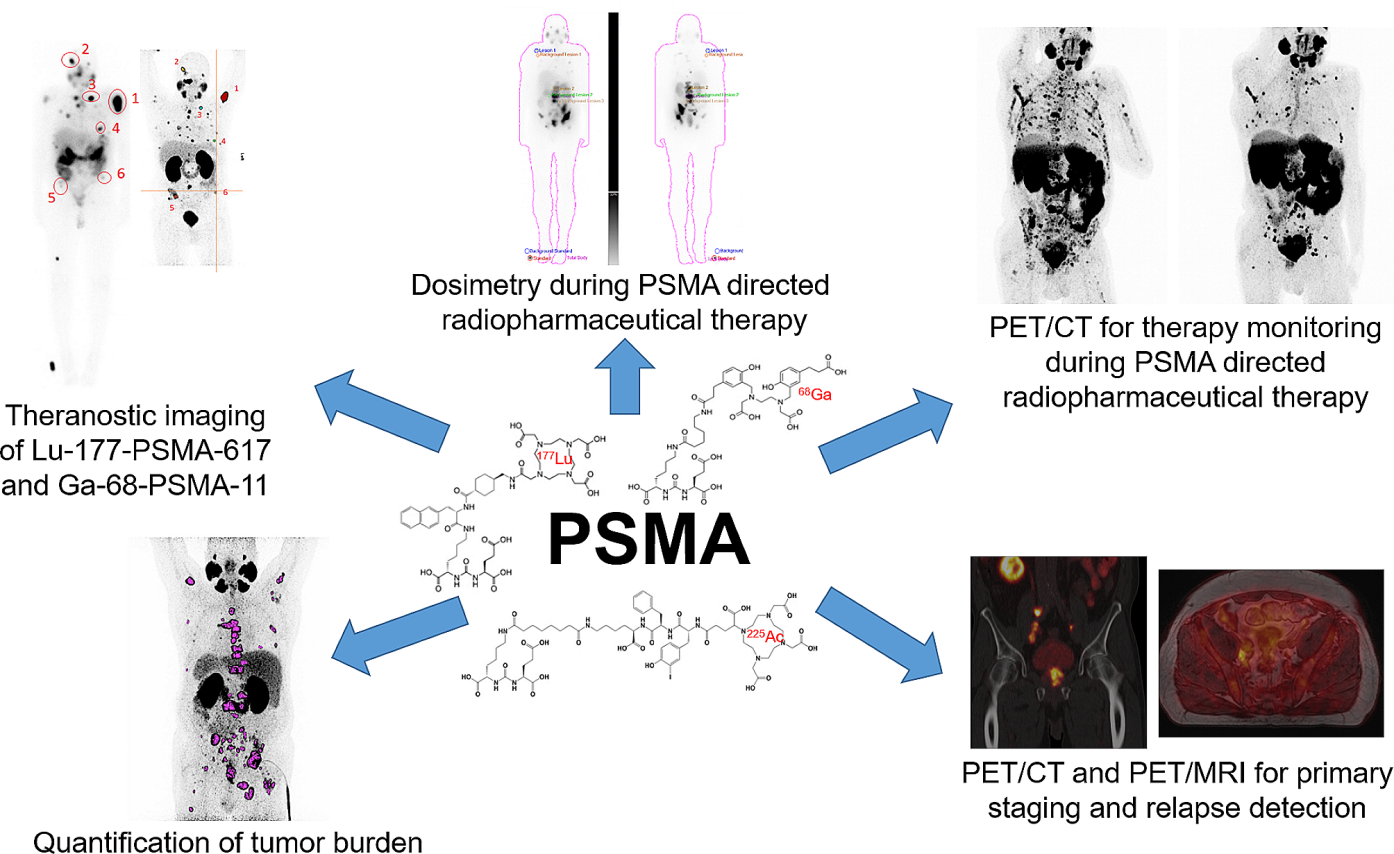
Overview of the wide range of applications of PSMA theranostics

Prostate-specific membrane antigen (PSMA), encoded by the *FOLH1* gene, has become a key target for diagnosing and treating PCa in all clinical stages and has proved particularly important for diagnosing and treating metastatic PCa. Low PSMA expression is one of the mechanisms that may lead to resistance to PSMA-based therapy (Fig. 6). Studies revealed that AR inhibition increases PSMA expression [81]. Therefore, this session highlights recent advances in PSMA theranostics in the current PCa therapeutic landscape.

#### Addressing the uncertainty in PSMA theranostic dose-responses

PSMA expression is associated with PCa aggressiveness, and ^177^Lu-PSMA-617 is approved as therapy in mCRPC. However, only a few dose-escalation studies have been performed, and despite numerous efforts, no dose-response curve for ^177^Lu-PSMA-617 has been established. Therefore, Dr. E. O’Neill addressed the importance of PSMA theranostic dose responses. He introduced the audience to studies investigating absorbed dose, standardised uptake value (SUV), or PSA_50_ value, which can be associated with total tumour volume and OS [24, 82, 83]. However, until now, due to tissue heterogeneity and differences in radiosensitivity, the dream of dosimetry and imaging-led guided treatment is still more of a mountain of work to come true. Therefore, he thinks a direct metric of the biological response to the radioligand therapy may overcome the lack of a predictive biomarker. γH2AX has been widely used to determine DNA damage and repair kinetics [84]. Moreover, preclinical and clinical studies have revealed that it can be used to image DNA damage and therapy response after radiation or radioligand therapy [85, 86]. To assess the repair capacity of each tumour and its response, he discussed the possibility of monitoring the DNA damage response caused by ^177^Lu-PSMA-617. He reported his studies using a dual-isotope single-photon emission computed tomography (SPECT) imaging strategy to monitor the change in the relationship between ^177^Lu-radioligand therapy and DNA damage (^111^anti-γH2AX-TAT) [87]. This dual-isotope SPECT imaging provided individualised tumour dose responses able to predict ^177^Lu-radioligand treatment efficacy and may be a potential method to predict response to radioligand therapy in PCa patients.

#### Alpha-therapy for PCa – aspects from radiochemical and nuclear-medical sight

An essential part of PSMA theranostics is the choice of the best radiopharmaceutical affecting imaging and therapy effectiveness. Therefore, Dr. M. Pretze discussed the usability of actinium (Ac) and lead (Pb) radiopharmaceutical in imaging and therapy. For ^225^Ac, he described efficient labelling of DOTA-conjugated peptides using an automated, GMP-compliant synthesis module. Innovations in chelators, such as macropa, allow for adequate antibody labelling even at room temperature [88]. Radiolabelled albumin-binding macropa-PSMA is currently in clinical translation from very promising preclinical results for both ^203/212^Pb and ^225^Ac. Although imaging can be further improved with higher doses, about one-third of patients show significant benefits, including extended life and improved palliative conditions, particularly among younger patients who may respond more favourably [89, 90]. Similarly ^203/212^Pb-VMT-α-NET facilitate effective imaging, and one-third of NET-patients benefit from extended life with a median increase of 8.5 months and enhanced palliative care [91]. These findings underscore the therapeutic potential of ^225^Ac and ^212^Pb in improving patient outcomes in radiopharmaceutical applications.

#### Developments in PSMA imaging

As the last speaker, Prof. M. Miederer provided an overview of the current PSMA imaging developments. PSMA PET has emerged as a significant tool in managing PCa, demonstrating its value in early diagnosis, therapy guidance, and relapse detection. In early diagnosis, Ga-68-PSMA PET combined with MRI shows high predictive value. Data from the PRIMARY trial showed that combining PSMA PET and MRI improved the negative predictive value and sensitivity for detecting clinically significant PCa in a population pre-screened with MRI [92]. In addition, results from the ProPSMA trial involving 251 patients with intermediate-to-high-risk prostate cancer (M_0_ stage) revealed that PSMA-PET-defined N_0_M_0_ patients had significantly longer freedom from treatment failure compared to N_1_M_0_ patients. At three years, 70% of N_0_M_0_ patients were free from treatment failure versus 46% of N_1_M_0_ patients, indicating the role of PSMA PET for early therapy guidance [93]. In addition, the data from Horsley and colleagues described the potential of PSMA PET for early detection and to map local recurrences after radical prostatectomy [94]. In the last part of his talk, he reminded the audience of the potential of PSMA PET for systemic treatment. The VISION trial could demonstrate that ^177^Lu-PSMA-617 therapy, combined with standard care, extended both imaging-based progression-free survival and OS in patients with advanced PSMA-positive metastatic CRPC [22]. He discussed the necessity for biomarkers to identify excellent therapy responses. He introduced the data from the biomarker analysis from the TheraP trial using the mean SUV of ^68^Ga-PSMA-11 and FDG as possible biomarkers for personalised medicine [24]. In mCRPC, high PSMA-PET SUVmean predicted a favourable response to ^177^Lu-PSMA-617 treatment. In contrast, high FDG-PET MTV was associated with lower responses, indicating that at least ^68^Ga-PSMA-11 could be used to identify a good responder patient group. Overall, he was highly optimistic about future developments and the use of PSMA theranostics, especially in current trials that show the combination of PSMA theranostics with ARPI and significantly improved progression-free survival [95, 96].

## Conclusion

At the “2nd International Androgen Receptor Symposium”, international participants from Austria, Belgium, Czechia, Finland, Germany, Netherlands, United Kingdom, and Northern Ireland discussed various aspects of AR research. They exchanged the latest findings to establish collaboration and improve patient care. Although the participants and speakers worked on various aspects of AR, they all agreed that better prognostic and diagnostic biomarkers are urgently needed to offer patients better and more personalised treatment in the future.

## Data Availability

Not applicable.

## Abbreviations

AR: Androgen receptor
ADT: Androgen deprivation therapy
Abi: Abiraterone
Ac: Actinium
Apa: Apalutamide
ARPI: Androgen receptor pathway inhibition
ARSi: Androgen receptor signalling inhibition
AR-V: Androgen receptor splice variant
CAF: Cancer-associated fibroblasts
CDK: Cyclin-dependent kinases
Daro: Darolutamide
DHT: Dihydrotestosterone
Doc: Docetaxel
dsRNA: Double-stranded RNA
Enza: Enzalutamide
FDG: Fluorodeoxyglucose
GnRH: Gonadotropin-releasing hormone
GR: Glucocorticoid receptor
HSP: Heat-shock proteins
mCRPC: Metastasised castration-resistant prostate cancer
mHSPC: Metastasised hormon-sensitive prostate cancer
NET: Neuroendocrine tumour
NHT: Novel hormonal therapy
Ola: Olaparib
Pb: Lead
PCa: Prostate cancer
PDX: Patient-derived xenografts
PET: Positron emission tomography
PKR: Protein kinase
PROTAC: Proteolysis targeting chimaera
PSMA: Prostate-specific membrane antigen
RLT: Radio ligand therapy
SAL: Supraphysiological androgen levels
SPECT: Single-photon emission computed tomography
SUV: Standardised uptake value
Talazo: Talazoparib
